# Identification and characterization of intestine microRNAs and targets in red swamp crayfish, *Procambarus clarkii* infected with white spot syndrome virus

**DOI:** 10.1371/journal.pone.0187760

**Published:** 2017-11-09

**Authors:** Zhi-qiang Du, Kai Wang, Xiu-li Shen, Yan-hui Jin, Hai-xia Jin, Xin-cang Li

**Affiliations:** 1 School of Life Science and Technology, Inner Mongolia University of Science and Technology, Baotou, Inner Mongolia autonomous region, China; 2 Library, Inner Mongolia University of Science and Technology, Baotou, Inner Mongolia autonomous region, China; 3 East China Sea Fisheries Research Institute, Chinese Academy of Fishery Sciences, Shanghai, China; Laboratoire de Biologie du Développement de Villefranche-sur-Mer, FRANCE

## Abstract

MicroRNAs (miRNAs) are small non-coding endogenous RNA molecules that play important roles in the innate immunity system of invertebrates, especially in the aspect of antivirus. In the present study, high-throughput small RNA Illumina sequencing systems were used to identify differentially expressed miRNAs (DEMs) from the intestines of *Procambarus clarkii* that were infected with white spot syndrome virus (WSSV). As a result, 39 known and 12 novel miRNAs were identified in both NG and WG small RNA libraries. Seven DEMs were determined to be involved in the antiviral innate immunity in the intestines of *P*. *clarkii*. The results of the target gene predictions of the DEMs showed that the putative target genes of these 7 DEMs are related to tight junctions, vascular smooth muscle contraction regulation of the actin cytoskeleton, focal adhesion, RNA transport, mRNA surveillance, viral carcinogenesis, and Salmonella infection. These results provide theoretical insights for future studies on the antiviral immunity of crustaceans.

## Introduction

*Procambarus clarkii* is frequently used as a model organism to study the molecular mechanisms of innate immunity in invertebrates [1]. *P*. *clarkii* has become one of the most important aquatic species in China due to its excellent disease resistance against pathogens [2]. Recent studies have fully examined antibacterial innate immunity in invertebrates, investigating scope from immunity-related genes to signaling pathways [3]. However, the studies about antiviral innate immunity should be strengthened.

White spot syndrome disease, which is caused by white spot syndrome virus (WSSV), often occurs in shrimp, crayfish, lobsters, and crabs [4]. A massive outbreak could bring huge economic losses for the crustacean breeding industry. However, effective treatments for white spot syndrome disease have not yet been developed. Therefore, it is necessary to perform additional studies concerning the defense and infection mechanisms between the host and WSSV using modern molecular biology techniques.

During the last decade, an important advancement in molecular biology was the discovery of small non-coding RNAs, including microRNAs, siRNAs, piRNAs, tncRNAs, and lncRNAs [5]. In recent years, microRNAs (miRNAs) have been extensively studied in the aspect of molecular immunity. Several miRNAs, which are endogenous, non-coding RNAs approximately 22 or 23 nucleotides in length, were originally found in eukaryotes [6]. Mature miRNAs are important post-transcriptional regulators that are generally present in animals and plants [7]. They also play important roles in cell differentiation, cell proliferation, immunity, autophagy, apoptosis, and signal transduction [8]. Recent studies reported that miRNAs are also involved in innate immunity in crustaceans, especially in antibacterial and antiviral immunity. For example, 195 miRNAs were predicted to participate in the hemocyte antibacterial innate immunity in *P*. *clarkii* infected with *Spiroplasma eriocheiris* [2]. In *Marsupenaeus japonicas* infected with *Vibrio alginolyticus*, 55 differentially expressed miRNAs (DEMs) were predicted to be involved in the hemocyte’s immunity response against bacterial infection [9]. In *M*. *japonicas* infected with WSSV, 63 miRNAs were determined to be involved in the lymphoid organs’ antiviral responses [10]. In the hematopoietic tissue of *Cherax quadricarinatus* infected with WSSV, 2 known miRNAs and 106 novel miRNAs were identified and predicted [7]. In *Litopenaeus vannamei* infected with WSSV, 37 miRNAs homologs were determined to participate in antiviral innate immunity in the hepatopancreas [11]. These abovementioned results could direct us to a new area of antiviral innate immunity research and may also aid in the establishment of new immunity-defending strategies against different pathogen infections in crustaceans.

In the present study, high-throughput sequencing was performed to comparatively analyze two small RNA libraries from the intestines of normal crayfish (NG) and WSSV-infected crayfish (WG). The differentially expressed miRNAs from the two libraries (NG and WG) were identified and the potential target genes of these DEMs were predicted. Several related signaling pathways were also identified. This study could help elucidate the role of miRNAs in regulating the innate immune response in crayfish and may also contribute to the development of new immune strategies for effective protection against WSSV infections in crustaceans.

## Materials and methods

### Ethics statement

The following experimental procedures comply with the current applicable laws of China, where they were performed. No specific permits were required for the research content in this article. Crayfish Individuals were maintained in appropriate laboratory conditions to guarantee their welfare and responsiveness. This study was also approved by the Zhejiang University in China.

### Immunity challenge

*P*. *clarkii* (approximately 15–20 g) were purchased from a commercial aquaculture market in Hangzhou, Zhejiang Province, China and were cultivated in water tanks at 26–28°C for 10 days to adapt to the surviving environment [1]. All crayfish were fed twice daily with artificial food throughout the entire experiment. For WSSV infection, WSSV (3.2 × 10^7^ copies per crayfish) was injected into the abdominal segment of each crayfish [12]. The intestines were collected 36 h after WSSV infection from ten WSSV-infected crayfish, which were termed the WSSV-infected group (WG). The intestines were also collected from ten normal crayfish, which were termed the normal group (NG). All intestines were frozen in liquid nitrogen and temporarily stored at -80°C until total RNA extraction [13].

### RNA extraction and quality analysis

The intestine samples of WG and NG crayfish were delivered to the Beijing Genomics Institute-Shenzhen (BGI, Shenzhen, China) for total small RNA extraction. Briefly, total small RNA from WG and NG was extracted using the *mirVana*^*™*^ micro-RNA Isolation Kit (Ambion, USA) according to the manufacturer’s protocol. The quality of small RNA samples treated with DNase I (Invitrogen, USA) was determined on a Nanodrop spectrophotometer (Nanodrop Technologies, USA). The RNA integrity number (RIN) was determined on an Agilent BioAnalyzer (Agilent Technologies, USA). RNAs with an RIN > 8.0 were chosen for small RNA library preparation and Illumina sequencing [7].

### Small RNA library preparation and Illumina sequencing

Approximately 1 μg of total small RNA from each sample was used as input for small RNA library preparation. In brief, small RNA libraries for WG and NG were produced using the NEBNext^®^ Multiplex Small RNA Library Prep Set for Illumina^®^ (NEB, USA) according to the manufacturer’s protocol. The quality of the small RNA library was determined on an Agilent Bioanalyzer 2100 system (Agilent Technologies, USA). Two qualified small RNA WG and NG libraries were sequenced on an Illumina HiSeq 2000 platform [14]. In the following analysis process, the default parameters were used.

### Data analysis and miRNA annotation

A filtering step was carried out to remove low-quality reads, including reads with 5' primer contaminants, reads without a 3' primer, reads with a poly(A) tail, reads without the insert tag, and reads shorter than 18 nt. The length distribution of the clean reads was then analyzed. At present, *P*. *clarkii* genomic data are not available. Besides, the relationship is relatively close between *P*. *clarkii* and *Daphnia pulex* in evolutionary level. In some articles about crustaceans’ microRNA sequencing, *D*. *pulex* genome was often chosen as the reference to analyze sequencing results [2, 7, 14, 15]. In present paper, the small RNA tags were mapped to the *D*. *pulex* genomic sequence using Bowtie 2 to analyze the expression level and distribution [2, 8]. The clean reads were subsequently analyzed using the Rfam 12.0 database to match the known small RNAs, including rRNAs, tRNAs, snRNAs, snoRNAs and other non-coding RNAs. According to sequence similarity, the remainder of the reads were classified into different categories and aligned to known and novel miRNAs for identification using the miRBase database (version 21.0) [16]. The MIREAP program was used to predict novel miRNAs from unannotated small RNAs. Based on specific positions of the miRNA hairpins, the characteristic hairpin structure of the miRNA precursor was used to predict novel miRNAs [17].

### Differential expression analysis of novel and known miRNAs

To confirm the differentially expressed genes between the WG and NG libraries, the miRNA expression levels were normalized to determine the expression in transcripts per million (TPM) using DESeq R package software version 2.0 [18]. Normalization was performed as follows: normalized expression = (actual miRNA count * 1,000,000/total count of clean reads). Finally, the fold-change and p-value were calculated and used as the threshold to determine significant differences between the differentially expressed genes [19].

### Target gene prediction of differentially expressed miRNAs

Because *P*. *clarkii* genomic data were not available, the transcriptome sequencing results from the intestines were used as the reference genome to perform target gene prediction. miRanda [20] and RNAhybrid (http://bibiserv.techfak.uni-bielefeld.de/rnahybrid/) software were used to predict miRNA target genes.

All miRNA targets were categorized into functional classes using GOseq and topGO software. KOBAS software (http://kobas.cbi.pku.edu.cn/home.do) was used to test the statistical enrichment of all miRNA target genes in KEGG pathways [21].

## Results and discussion

### Data analysis and length distribution of small RNAs

**Fig 1:**
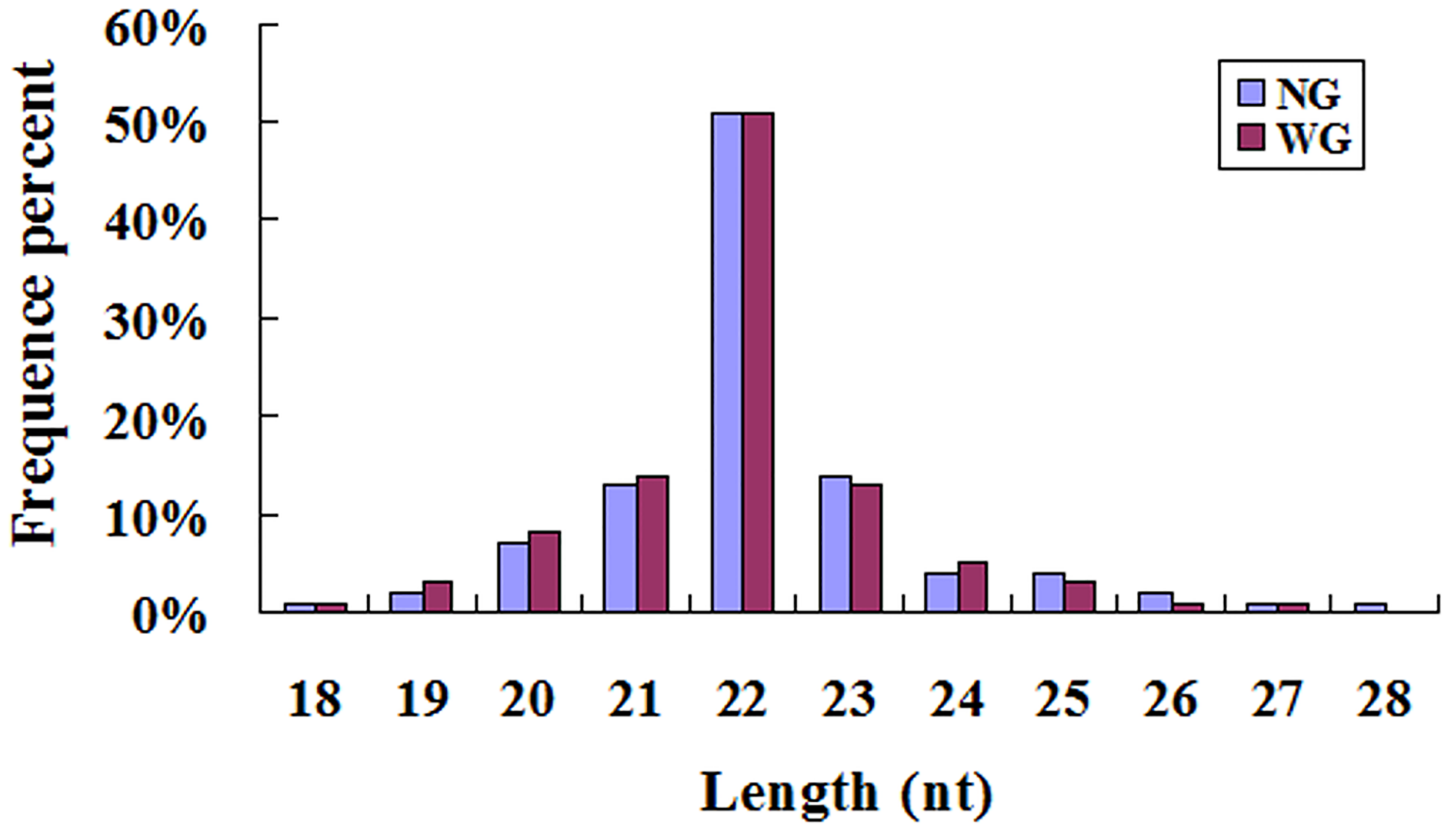
Length distribution of small RNAs sequences in NG and WG library.

Illumina HiSeq 2000 high-throughput small RNA sequencing yielded 11,857,305 and 11,126,798 raw reads from the NG and WG small RNA libraries, respectively. The number of clean reads in the NG and WG libraries was 10,281,968 (86.71%) and 9,608,326 (86.35%), respectively. The length distributions of the clean reads in the NG and WG libraries were summarized and show that most small RNAs in both NG and WG small RNA libraries are 22 nt in length. The remainders of the small RNAs were 23 nt and 21 nt in the NG and WG libraries (Fig 1). Generally speaking, the length of small RNA was between 18 nt and 30 nt. And miRNA was normally 21 nt or 22 nt. Our results conform to this pattern and are consistent with the previous reports on microRNA libraries of the hematopoietic tissue of *C*. *quadricarinatus* [7], the hemocytes of *M*. *japonicas* [9], and the hepatopancreas of *L*. *vannamei* [11].

### Identification and annotation analysis of miRNAs

The alignment of unique sequences against non-coding RNAs in the GenBank and Rfam (version 12.0) databases revealed five types of specific small RNAs, including rRNAs, tRNAs, miRNAs, snRNAs, and snoRNAs. Among them, miRNAs accounted for 38.15% and 39.82% of the total sequences in the NG and WG libraries, respectively, and they accounted for 8.41% and 7.62% of the unique sequences in the NG and WG libraries, respectively. Compared with the corresponding results of earlier work with red swamp crayfish [14], some differences were found. According to their results, miRNAs accounted for 2.79% and 2.40% of unique sequences in control and trial small RNA libraries, respectively. Our results about the proportion of miRNAs to unique sequences were obviously higher. Next, a closely related organism was chosen to perform miRNA prediction using the abovementioned miRNA sequences.

### Nucleotide bias analysis of identified miRNAs in both NG and WG miRNAs libraries

**Fig 2:**
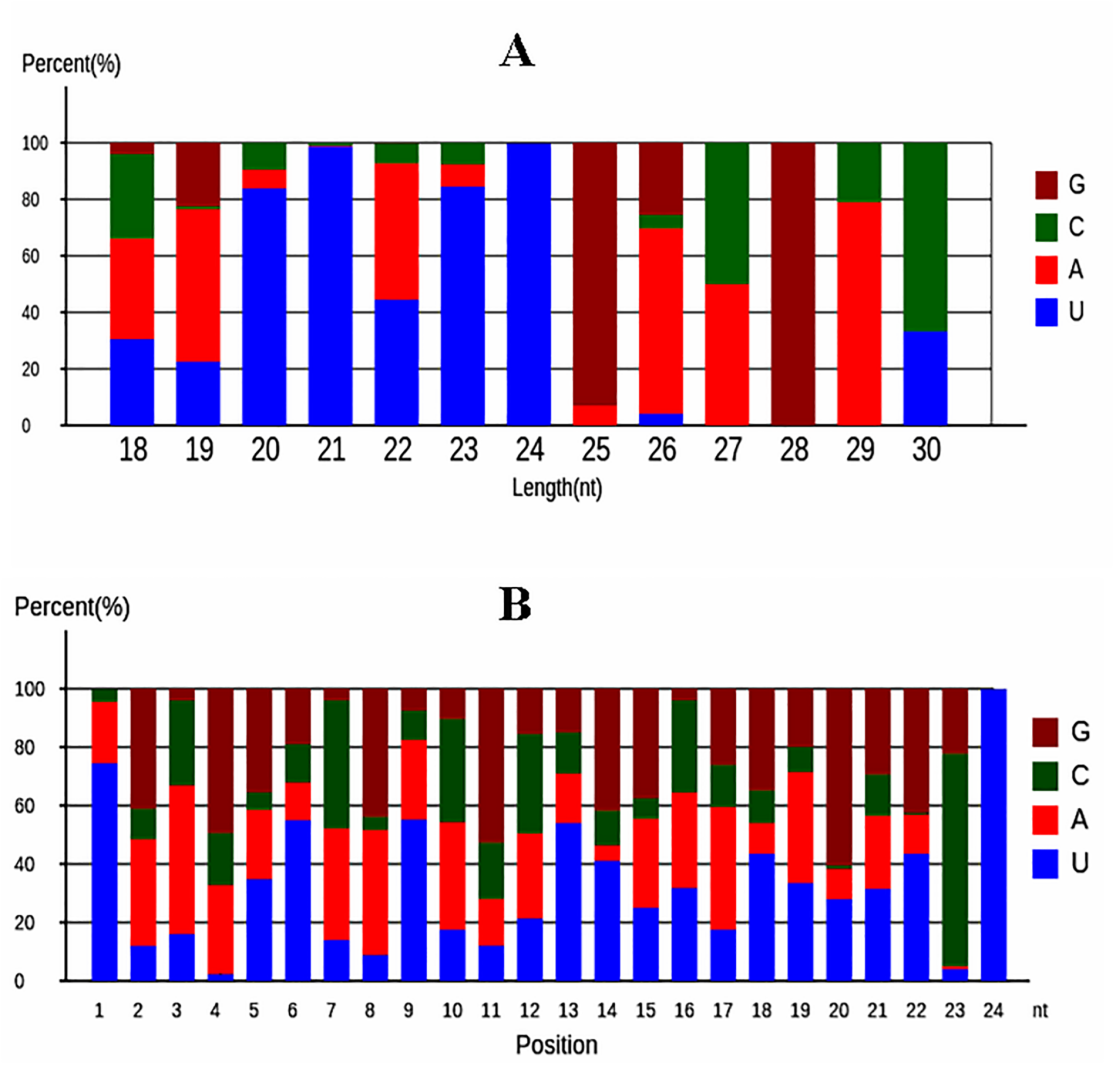
First nucleotide bias (A) and position nucleotide bias (B) analysis for miRNAs.

The structure of miRNAs possessed sequence specificity. In mature miRNA, the position between 2 to 8 bp was called seed region, which was highly conserved [22]. And it had a strong preference for pyrimidine residues, uracil in particular. This phenomenon suggested that uracil might have important roles in biological functions of miRNAs [23]. In present paper, the nucleotide bias at the first position and the percentage of four nucleotides (A, G, C, and U) appearing at each position in miRNAs were analyzed (Fig 2A and 2B). The results showed that a dominant bias to uracil (U) at the first nucleotide, especially the miRNAs with a length of 20–24 nt (Fig 2A). The results of percentage analysis of four nucleotides (A, G, C, and U) appearing at each position showed that the most dominant bias to U were the 1st, 6th, 9th, 13th, 14th, 18th, 22th, and 24th nucleotides (Fig 2B). These two biases of nucleotides in miRNAs in both NG and WG libraries were quite similar with those results reported in previous articles [24].

### Prediction of known and novel miRNAs

**Fig 3:**
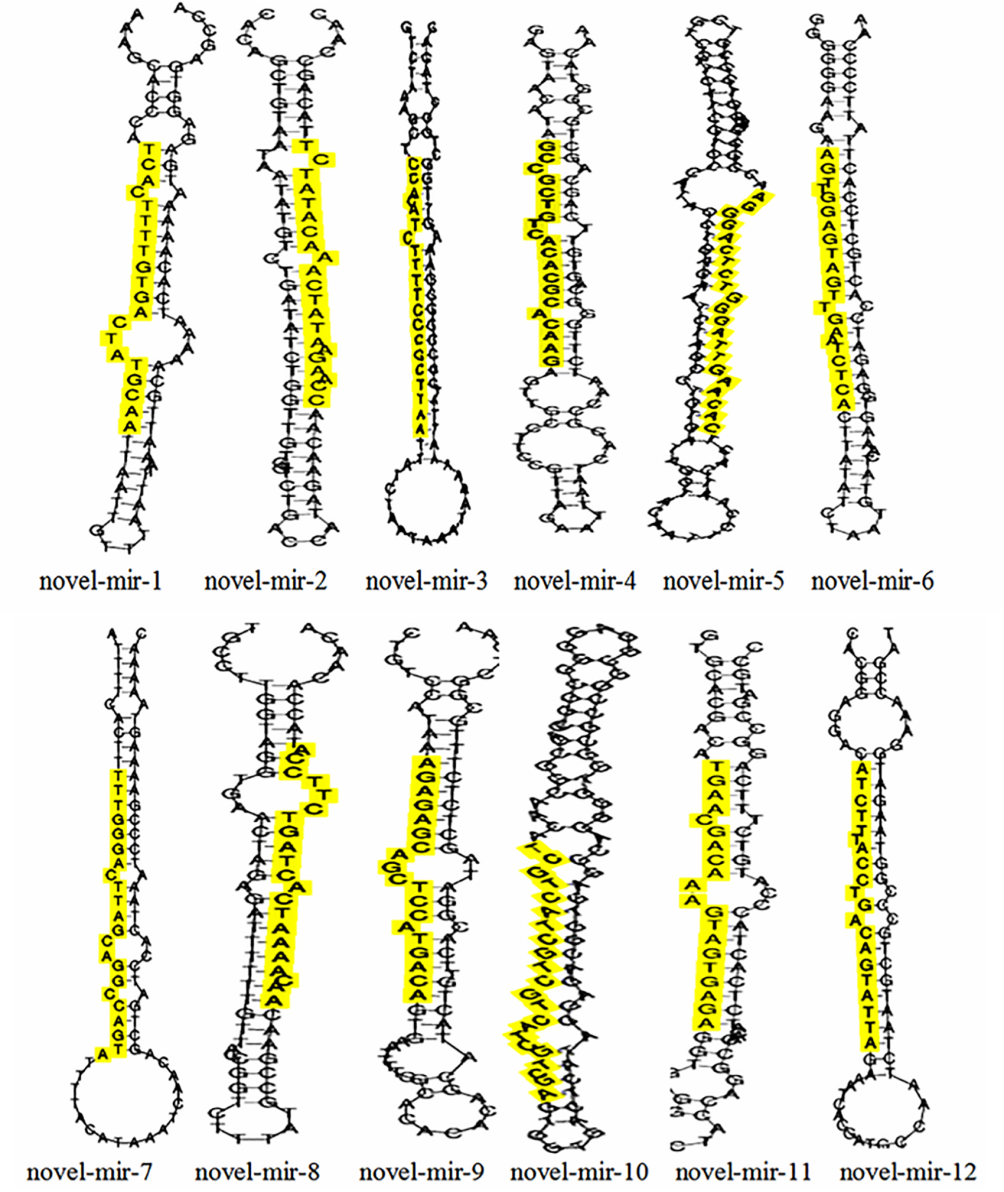
Putative folding structure of predicted novel miRNA precursors in NG and WG libraries. The yellow shading regions in each structure delegated the mature predicted novel miRNA sequence.

To identify known miRNAs in the crayfish intestine miRNA libraries (NG and WG), mappable sequences were aligned to the miRNA sequences of *Daphnia pulex* in the miRBase database (version 21.0). In total, 3,492 and 3,073 miRNAs were annotated in the NG and WG libraries, respectively. Among them, 39 known miRNAs were identified in both NG and WG libraries, 38 of which were identified in both NG and WG libraries simultaneously. Dpu-miR-31 was only identified in the NG library. The counts and sequences of these known miRNAs are shown in Table 1. Twelve novel miRNAs were identified in both NG and WG libraries (Table 2). Among them, 8 were predicted and identified in the NG library and 9 were predicted and identified in the WG library. The sequences and expression levels of these 12 novel miRNAs are shown in Table 2. Among them, novel-miR-1, novel-miR-2, novel-miR-3, novel-miR-4, novel-miR-5, novel-miR-6, novel-miR-7, and novel-miR-8 were identified in the NG library; and novel-mir-1, novel-mir-2, novel-mir-4, novel-mir-6, novel-mir-8, novel-mir-9, novel-mir-10, novel-mir-11, and novel-mir-12 were identified in the WG library. The stem-loop structures of the abovementioned 12 novel miRNAs were also predicted and are shown in Fig 3.

**Table 1:** Known miRNAs identified in NG and WG libraries.

No.	miRNA ID	Sequences (5’-3’)	Reads in NG library	Reads in WG library
1	Dpu-bantam	TGAGATCATTGTGAAAGCTGATT	2635	2127
2	Dpu-miR-1	TGGAATGTAAAGAAGTATGGAG	3311659	3379715
3	Dpu-miR-10	TACCCTGTAGATCCGAATTTGT	2794	4536
4	Dpu-miR-100	AACCCGTAGATCCGAACTTGTGT	35277	31765
5	Dpu-miR-12	TGAGTATTACATCAGGTACTGGT	10090	7423
6	Dpu-miR-124	TAAGGCACGCGGTGAATGCCAAG	122	56
7	Dpu-miR-133	TTGGTCCCCTTCAACCAGCTGT	739	971
8	Dpu-miR-137	TATTGCTTGAGAATACACGTTG	29	26
9	Dpu-miR-153	TTGCATAGTCACAAAAGTGATG	199	155
10	Dpu-miR-193	TACTGGCCTGCTAAGTCCCAAA	575	690
11	Dpu-miR-2	TATCACAGCCAGCTTTGATGAGC	3962	3359
12	Dpu-miR-252a	CTAAGTACTCGTGCCGCAGGAG	15	11
13	Dpu-miR-252b	CTAAGTAGTAGTGCCGCAGGTA	40759	37940
14	Dpu-miR-263a	AATGGCACTGGAAGAATTCAC	6184	4562
15	Dpu-miR-263b	CTTGGCACTGGAAGAATTCACA	302	269
16	Dpu-miR-275	TCAGGTACCTGAAGTAGCGCGCG	233216	140146
17	Dpu-miR-276	TAGGAACTTCATACCGTGCTCT	24780	20165
18	Dpu-miR-278	TCGGTGGGACTTTCGTCCGTGT	39	38
19	Dpu-miR-279a	TGACTAGATCCACACTCATCCA	40210	34342
20	Dpu-miR-281	TGTCATGGAGCTGCTCTCTTTAT	18	31
21	Dpu-miR-285	TAGCACCATTGGAATTCAGTTT	55	45
22	Dpu-miR-305	TTTGTACTTTATCAGGTGCTCT	880	703
23	Dpu-miR-307	TCACAACCTCCTTGAGTGAG	115	104
24	Dpu-miR-31	AGGCAAGATGTCGGCATAGCTGA	1	0
25	Dpu-miR-315	TTTTGATTGTTGCTCAGAAAGC	33960	22171
26	Dpu-miR-317	TGAACACAGCTGGTGGTATCTCAGT	12697	9491
27	Dpu-miR-34	TGGCAGTGTGGTTAGCTGGTTGTG	25056	23360
28	Dpu-miR-7	TGGAAGACTAGTGATTTTGTTGT	767	524
29	Dpu-miR-71	TGAAAGACATGGGTAGTGAGATG	42652	35320
30	Dpu-miR-745	GAGCTGCCCAGTGAAGGGCTTT	3	2
31	Dpu-miR-8	TAATACTGTCAGGTAAAGATGTC	44604	32741
32	Dpu-miR-92	AATTGCACTCGTCCCGGCCTGC	4	6
33	Dpu-miR-9-3p	ATAAAGCTAGGTTACCAAAGTTA	459	309
34	Dpu-miR-9-5p	TCTTTGGTTATCTAGCTGTATGA	5766	4354
35	Dpu-miR-965	TAAGCGTATGGCTTTTCCCCTG	970	1011
36	Dpu-miR-981	TTCGTTGTCGACGAAACCTGCA	8	1
37	Dpu-miR-993	GAAGCTCGTTTCTACAGGTATCT	3	2
38	Dpu-miR-iab-4-3p	CGGTATACCTTCAGTATACGTAAC	13	6
39	Dpu-miR-iab-4-5p	ACGTATACTGAATGTATCCTGA	22	13

**Table 2:** Novel miRNAs identified in NG and WG libraries.

No.	miRNA ID	Sequences (5’-3’)	Reads in NG library	Reads in WG library
1	novel-mir-1	TGCAAAAATCACAAAAATGAG	216	141
2	novel-mir-2	CCAAGAATATCAAACATATCT	9747	5906
3	novel-mir-3	CCACATCTTTTCCCGCTTAA	42	0
4	novel-mir-4	GCCGCTGTCACACGCACAAG	35	42
5	novel-mir-5	CACAAGTTAGGGTCTCAGGGA	32	0
6	novel-mir-6	AGTTGGAGTAGTTGAATCTCA	169	98
7	novel-mir-7	TTTGGGACTTAGCAGGCCAGTA	8	0
8	novel-mir-8	AACAAAATCACTAGTCTTCCA	10	7
9	novel-mir-9	TGTTCACGGATAGCTCTCTT	0	3628
10	novel-mir-10	TCGTCATCGTCGTCATCGTCGA	0	5
11	novel-mir-11	CTCACTACCCATGTCTTTCA	0	506
12	novel-mir-12	ATCTTTACCTGACAGTATTA	0	256

Further analysis of these 51 miRNA structures and functions could elucidate the roles of miRNAs in antiviral innate immunity in crustaceans and aid in the identification of their target genes. Moreover, the relationship between miRNAs and their target genes could clarify the interactions between signaling pathways that are regulated by miRNAs.

### Differentially expressed miRNA analysis and target gene prediction

High-throughput sequencing was a powerful method for providing important information about miRNA expression patterns by testing the sequencing frequency of miRNAs [25]. To confirm the changing situation of identified miRNAs between NG and WG libraries after WSSV infection, differential expression analysis was performed. Based on the sequencing data of the abovementioned 51 miRNAs, 7 differentially expressed miRNAs (DEMs) were identified between the NG and WG libraries. Comparison of the miRNA expression profiles revealed 3 miRNAs, novel-mir-9, novel-mir-11, and novel-mir-12, that were significantly up-regulated, and 4 miRNAs, Dpu-miR-124, novel miR-3, novel miR-5, and novel miR-7, that were significantly down-regulated in the WG small RNA library. All DEMs are shown in Table 3.

**Table 3:** Differential expression analysis for miRNAs between NG and WG libraries.

No.	miRNA ID	Reads in NG library	Reads in WG library	log2 Fold (WG/NG)	p value
1	Dpu-miR-124	122	56	-1.09 (down)	1.06E-06
2	novel-mir-3	42	0	-6.22 (down)	2.00E-11
3	novel-mir-5	32	0	-5.83 (down)	4.35E-09
4	novel-mir-7	8	0	-3.83 (down)	0.006377
5	novel-mir-9	0	3628	12.99 (up)	0.001
6	novel-mir-11	0	506	10.15 (up)	4.10E-84
7	novel-mir-12	0	256	9.17 (up)	1.43E-49

Because the *P*. *clarkii* genomic sequence is not available in the GenBank database, the *D*. *pulex* genomic sequence was used as the reference genome for target gene prediction of the abovementioned 7 DEMs. The related 3'-UTR sequences were determined using TargetScan and the miRanda database (version 21.0) [26]. The highest scoring target genes for these 7 DEMs (Dpu-miR-124, novel miR-3, novel miR-5, novel miR-7, novel miR-9, novel miR-11, and novel miR-12) were clotting protein precursor, HERC2-like E3 ubiquitin-protein ligase, dual oxidase, notch protein, Dicer 2, NADPH oxidase, and dedicator of cytokinesis protein 1-like isoform 2, respectively. Most of the putative target genes (e.g., clotting protein precursor, HERC2-like E3 ubiquitin-protein ligase, notch protein, and Dicer 2) are related to immunity. These results indicate that Dpu-miR-124, novel miR-3, novel miR-7, and novel miR-9 participate in the anti-WSSV immunity response. These predicted putative target genes may aid in a better understanding of the roles of the DEMs in antiviral innate immunity in crayfish.

### GO enrichment and KEGG pathways analysis of DEM target genes

**Fig 4:**
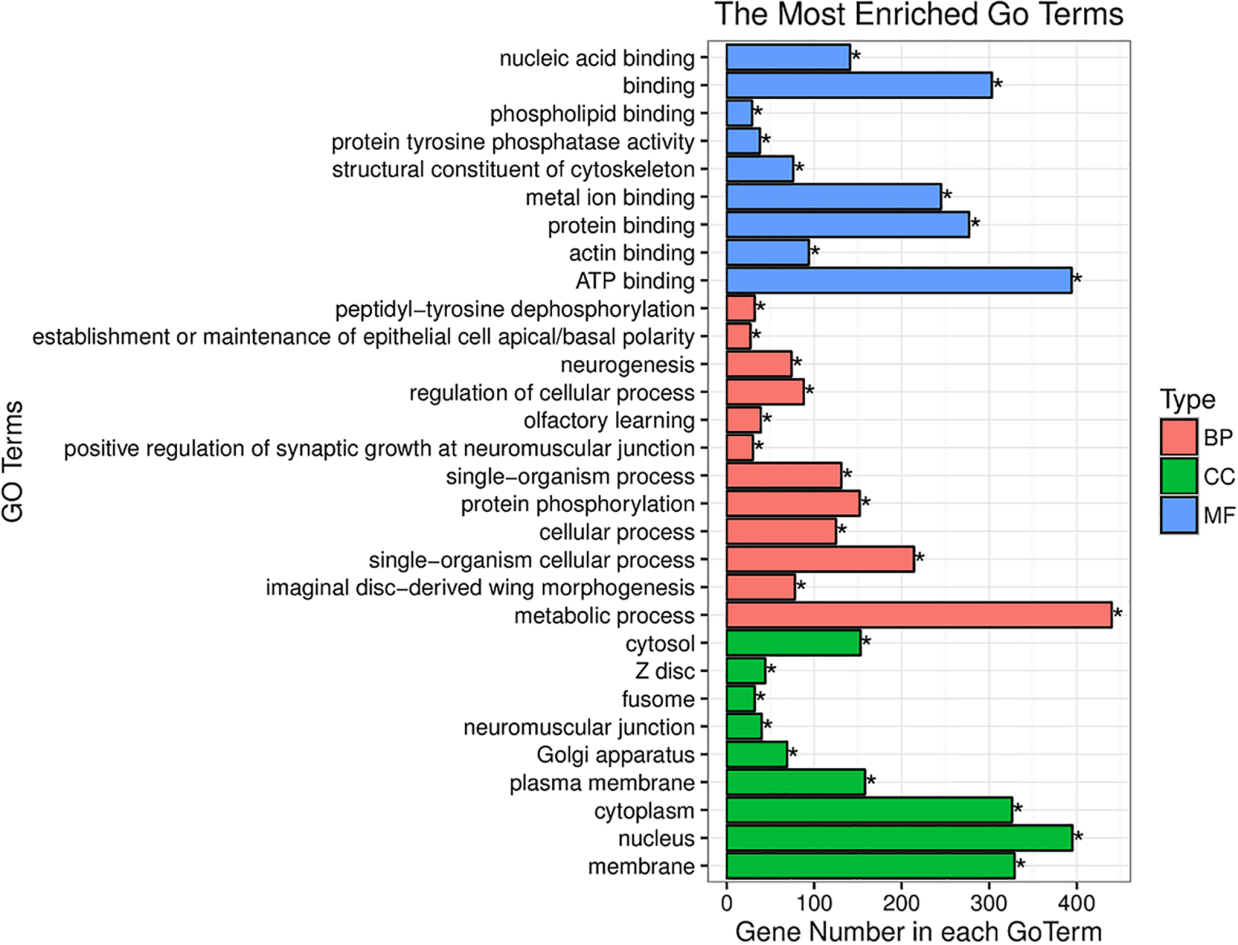
GO analysis of target genes of differently expressed miRNAs.

Following target gene prediction of the abovementioned 7 DEMs, 5,362 putative predicted target genes were identified and categorized into three classes by Gene Ontology (GO) enrichment analysis: cellular component (CC), molecular function (MF) and biological process (BP) [27]. In the cellular component class, the putative target genes were mostly related to the nucleus, membrane, and cytoplasm. In the molecular function class, the putative target genes were mostly related to ATP binding, binding, protein binding, and metal ion binding. In the biological process class, the putative target genes were mostly related to metabolic processes, single-organism cellular processes, and protein phosphorylation (Fig 4).

**Fig 5:**
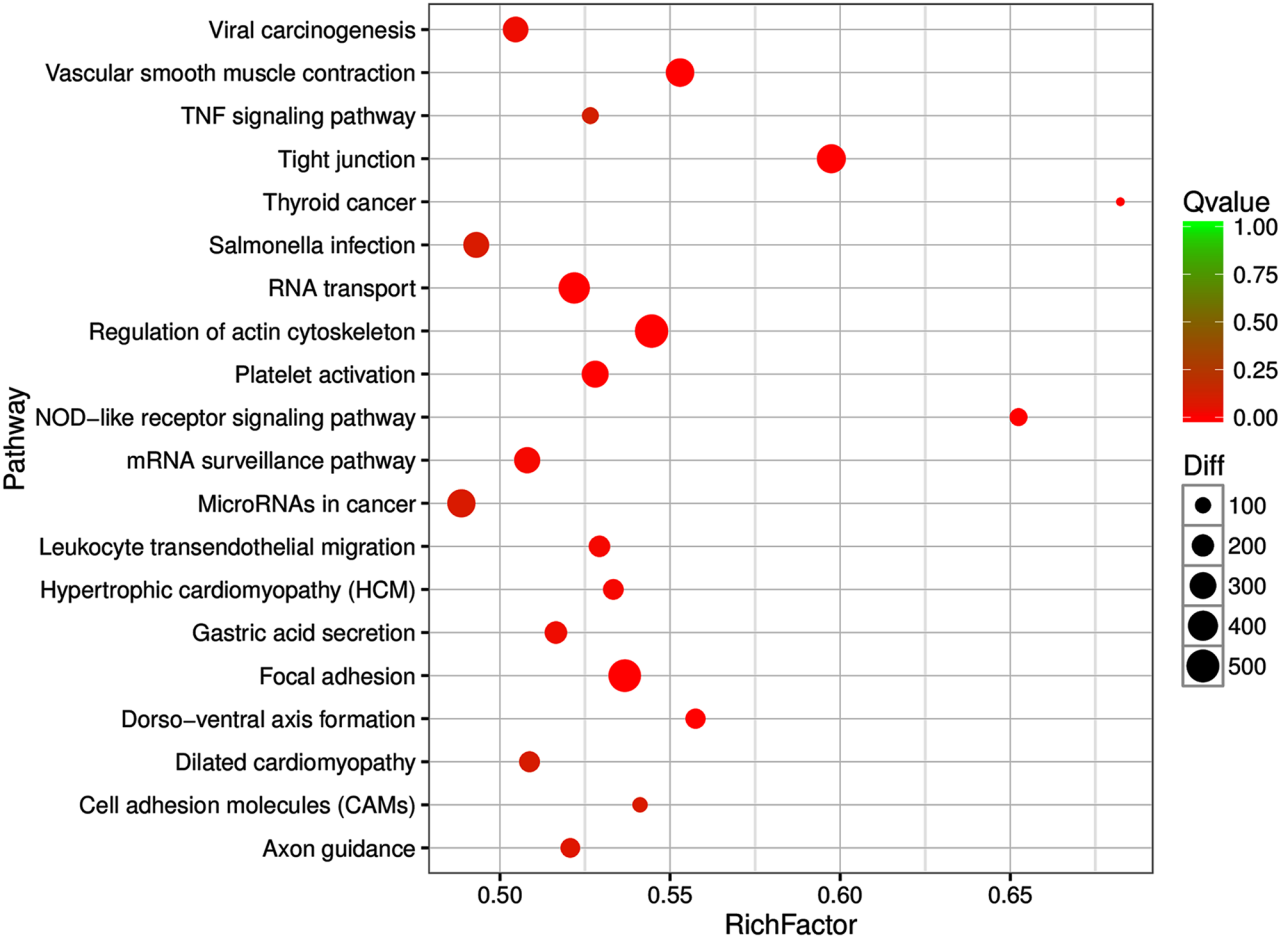
KEGG analysis for the target genes of six DEMs.

To fully elucidate the functions of the abovementioned 7 DEMs, KEGG pathway analysis was performed. The putative target genes were mostly enriched in tight junctions, vascular smooth muscle contraction regulation of the actin cytoskeleton, focal adhesion, RNA transport, mRNA surveillance, viral carcinogenesis, and Salmonella infection (Fig 5). In the future, it will be important to functionally validate these differentially expressed miRNA target genes.

## Conclusions

In conclusion, a total of 3,492 and 3,073 miRNAs were obtained from the NG and WG libraries, respectively, using the miRBase database (version 21.0). Among them, 39 known miRNAs and 12 novel miRNAs were identified. Subsequently, 7 DEMs were identified in the intestines of *P*. *clarkii* infected with WSSV. The putative target genes of these 7 DEMs are related to tight junctions, vascular smooth muscle contraction regulation of the actin cytoskeleton, focal adhesion, RNA transport, mRNA surveillance, viral carcinogenesis, and Salmonella infection. These results provide a theoretical foundation for future studies on antiviral innate immunity in crustaceans.
